# Surface treatments and coatings of hybrid glass ionomer cement to improve mechanical and physical properties

**DOI:** 10.1038/s41598-025-30202-2

**Published:** 2025-12-08

**Authors:** G. Zambon, M. G. Cagetti, S. Cirio, G. De Bortoli, A. Allam, A. C. Ionescu

**Affiliations:** 1 Department of Biomedical, Surgical, and Dental Sciences, School of Pediatric Dentistry, University of Milan, Via Beldiletto, 1/3, 20142 Milan, Italy; 2 Oral Microbiology and Biomaterials Laboratory, Department of Biomedical,Surgical, and Dental Sciences, University of Milan, Via Pascal 36, 20133 Milan, Italy; 3 Fondazione IRCCS Cà Granda Ospedale Maggiore Policlinico, Via della Commenda 10, 20122 Milan, Italy

**Keywords:** Health care, Materials science, Medical research

## Abstract

This in vitro study evaluated the flexural strength, fluoride release, water sorption, and solubility of a high-viscosity hybrid glass ionomer cement (HVGIC, Equia Forte HT, GC Europe) over 28 days following six surface treatments: Equia Forte Coat (light-cured, 20s), bonding agent (Clearfil SE Universal Bond, 20s), light-curing alone as thermal treatment (20s or 60s), petroleum jelly, and untreated control. Specimens were stored in artificial saliva at 37 °C. Flexural strength (three-point bend test, ISO 4049:2019) and fluoride release were assessed at 24, 48, 96 h and 28 days. Sorption and solubility were measured at 28 days. Statistical analysis included bivariate tests, Kaplan-Meier survival curves, Tukey’s post-hoc, and Weibull regression.

Proprietary coat and bonding agent reached the minimum required strength (MRS = 80 MPa) fastest (< 2 days), followed by petroleum jelly (2.5 days), 60s light curing (3 days), 20s (4 days), and control (5.5 days). Control showed the highest fluoride release initially, while at 28 days, 60s light-curing released the most fluoride. Proprietary coat and bonding agent showed minimal release. No significant differences in water sorption or solubility were found.

These findings suggest that specific coatings or prolonged light curing can improve HVGIC performance and longevity of restorations.

## Introduction

First introduced in 1972^1^, glass-ionomer cements (GICs) are still widely used in restorative dentistry. Application of GIC include full restorations for both primary and permanent teeth, fissure sealants, liners and bases, luting agents, and adhesives for orthodontic brackets^2^. Their properties of simple handling, good biocompatibility to pulp and surrounding tissue^3^, downregulating bacterial acidic metabolism^4^, adhesion to the tooth surface^5^, and fluoride release & recharge^6,7^ have contributed to their popularity.

All GICs are the result of a chemical setting reaction that occurs between glass ions (silica, alumina, fluoride, aluminum fluoride, strontium, together with minimal percentages of sodium, calcium, and others) and an aqueous solution of carboxylic acids. Upon mixing the liquid and powder components, a rapid acid-base reaction is initiated, lasting approximately 2–10 min and resulting in the formation of an ionically crosslinked polysalt matrix^8^. Subsequently, a secondary acid-base reaction phase ensues, characterized by the gradual release of cations (mainly calcium, aluminum, strontium) into the matrix, which continues for up to 24 h^9^. During the initial setting phase, the material exhibits high susceptibility to water sorption. Early contact with moisture causes loss of ions from the surface of the material, leading to surface erosion^10^. In the following maturation phase, the material becomes particularly vulnerable to dehydration. These moisture-related sensitivities can adversely affect the material’s mechanical properties, resulting in decreased flexural strength and increased wear of the restorations^11,12^. Also, water loss can cause microcracks, volume variations and adhesion deficiency.

More modern GIC restorative materials, including hybrid GICs, incorporate finer, highly-reactive glass particles and an increased length of the polyacrylic acid chains^13^. This formulation optimized the mechanical performance of the material, improving the well-known drawbacks of the material such as its flexural strength, wear resistance, and aesthetic properties^14–16^. Even so, the long-term mechanical performance of hybrid GICs remains significantly inferior to that of resin-based composites, particularly in terms of durability and resistance to occlusal stresses^17^.

To overcome such shortcomings of all GICs, several techniques were proposed by clinicians with the common aim of providing a protective effect from extrinsic fluids, while enabling complete maturation of the material in a more controlled environment. In fact, delayed exposure to oral fluids has been shown to reduce the formation of cracks and porosity in the restoration^18^. There is limited literature proposing to protect the GIC surface from the oral environment by insulating the restoration surfaces with various agents, such as varnishes, light-cured bonding resins, petroleum jelly (solid or liquid) and nail varnishes^18–20^. Following this approach, some manufacturers recommended the application of a light-cured, self-adhesive protective coating such as a light-cured bonding resin. The coating was specifically optimized to work in conjunction with the material, for instance, incorporating nanofiller particles, thus also improving wear resistance and aesthetics^13^. Nevertheless, the application of bonding resins, varnishes, or other surface coatings is not primarily intended to directly enhance the mechanical performance of glass ionomer cements (GICs) by providing a reinforcing layer. Instead, their main function is to create an insulating barrier that prevents undesirable water and ion exchange during the critical maturation phase of the material, thereby ensuring optimal setting and stability over time^21^. From a clinical standpoint, both manufacturer-provided coatings and bonding agents are tasteless once polymerized, minimizing patient discomfort. Similarly, petroleum jelly is devoid of any discernible flavor, although its gelatinous consistency may be perceptible during the initial moments following application.

Some studies have suggested the application of a light-curing device to be used as a thermal energy source to accelerate the setting rate of GICs and hasten their maturation phase, thus achieving better mechanical proprieties^22–24^. At molecular level, polyacrylic acid attacks the glass particles, releasing cations (Al^3+^ and Ca^2+^) that form ionic bridges with carboxyl groups, initiating matrix cross-linking. During the setting reaction, aluminum transitions from a tetrahedral (Al^4^) to a more stable octahedral coordination (Al^6^), contributing to the maturation of the structure^25^. Studies have demonstrated that increased kinetic energy, provided as heat, accelerates this structural transition, increasing the concentration of Al^6^ and, consequently, the microhardness of the material^23,26^. However, to the authors’ knowledge, there is no comprehensive study comparing at the same time the efficacy of the mentioned techniques in improving the material characteristics.

The aim of this set of experiments was to compare the flexural strength, water sorption and solubility, and fluoride release of a hybrid glass ionomer cement (HGIC, Equia Forte HT, GC Europe, Luzern, Switzerland) subjected to different treatments spanning the most used coating techniques described in the literature, namely:

- coated on all surfaces with Equia Forte Coat and light-cured for 20 s at 1100 mW/cm^2^;
- coated on all surfaces with a bonding agent (Clearfil Universal Bond Quick, Kuraray Noritake, Tokyo, Japan) and light-cured for 20 s at 1100 mW/cm^2^;
- covered on all surfaces with petroleum jelly;
- light irradiated for 60 s at 1100 mW/cm^2^ to provide thermal energy during the setting reaction of the material.

A control group was additionally introduced to exclude the influence of a thermal effect of light-curing on resin coated specimens and was light irradiated for 20 s at 1100 mW/cm^2^.

The main control group was left to cure without any surface treatment or light-curing application.

## Results

The light-curing of the specimens produced an increase in their temperature as a consequence of the thermal irradiation. Light irradiation at 1100 mW/cm^2^ produced an increase of 8.9 (± 0.7 SD) and 24.2 (± 4.9) °C after 20 s and 60 s, respectively, confirming a significant thermal effect of the application of the light source during the material initial setting. Interestingly, mixing procedures increased the material temperature by 1.8 (± 0.9 SD) °C compared to room temperature.

### Flexural strength

**Fig. 1:**
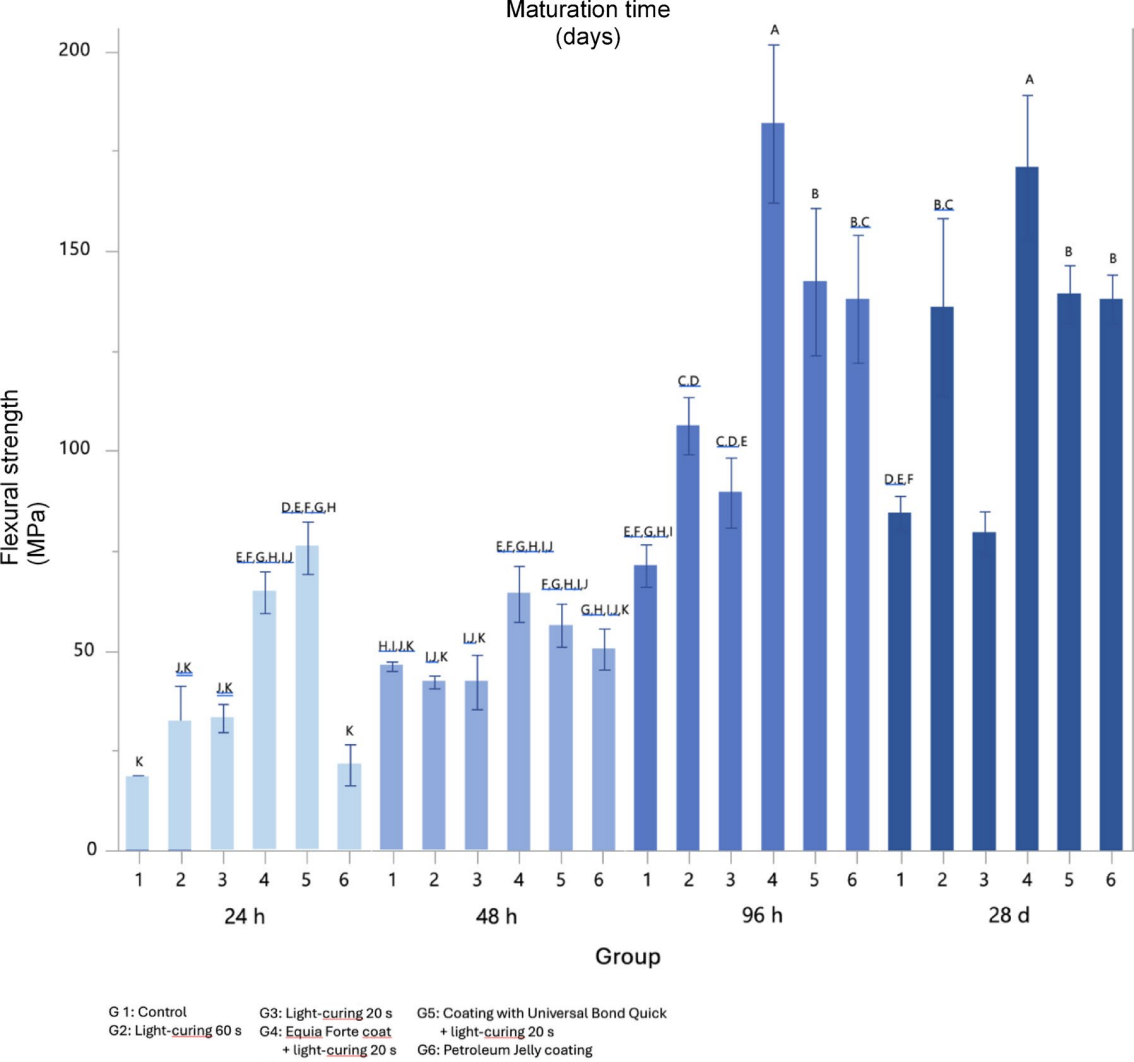
Flexural strength values for each group at 1, 2, 4 and 28 days. Mean MPa ± 1 standard error are displayed, while different superscript letters indicate a significant difference as assessed by Tukey’s, *p* < 0.05.

Flexural strength data (mean values and standard deviations, obtained by 3-point bend test according to ISO 4049:2019) are summarized in Fig. 1. At 24 h, a statistically significant difference was found between the groups treated with Equia Forte Coat (64.6 MPa) and bonding agent (75.8 MPa) and the other groups (*p* < 0.05), with untreated specimens showing the lowest strength values (18.2 MPa). At 24 and 48 h, all groups showed strength values below the minimum required strength (MRS, 80 MPa). A significant increase in flexural strength values at 96 h was observed in the treated groups compared to the control (*p* < 0.05). The highest mean values were observed in the Equia Forte Coat (181.9 MPa) and universal bonding agent (142.3 MPa) groups, followed by petroleum jelly (138.1 MPa) and 60 s light cured (106.3 MPa) groups. All groups, except the control, exceeded MRS. At 28 days, the values of each group remained stable, showing no statistically significant variations compared to the previous time point (*p* > 0.05).

**Fig. 2:**
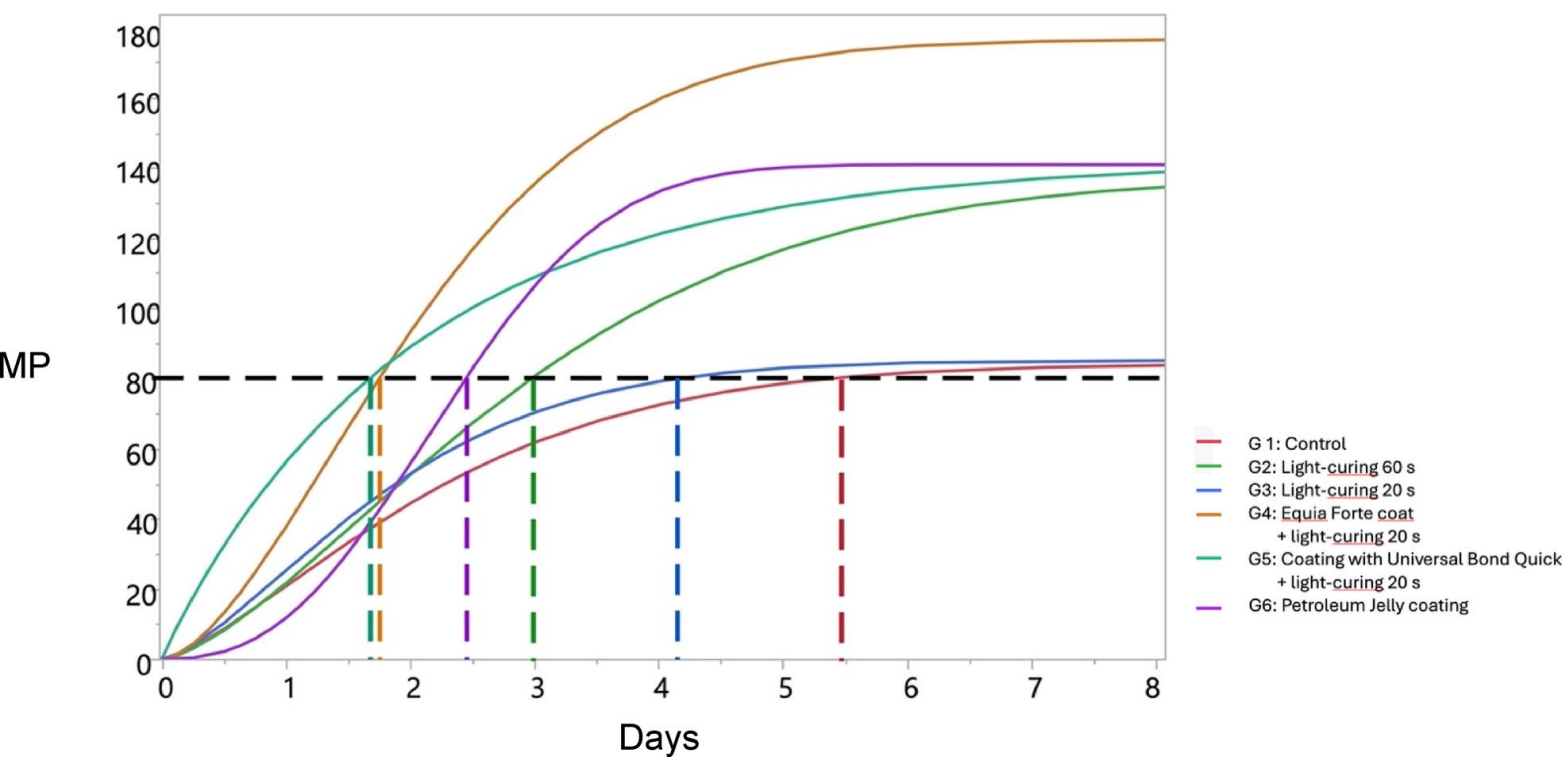
The Weibull diagram displaying the sigmoid regression fit of each group’s flexural strength distribution (MPa) in relation to the maturation time (days).

The Weibull diagram describing the sigmoid regression model of each group’s value distribution in relation to time (days) is presented in Fig. 2. According to the model, without any treatment, the material reaches values exceeding MRS in approximately 5.5 days. In contrast, surface treatments achieve this threshold more rapidly: light cured for 60 s within 3 days, petroleum jelly within 2.5 days, and Equia Forte coat and bonding agent in less than 2 days.

Specimens to which the coat was applied only on top or bottom had a strength of 28.2 and 33.7 MPa, respectively. These values were not significantly different to the untreated control (*p* = 0.89 and *p* = 0.30, respectively), and significantly lower than the specimens treated on all surfaces (*p* < 0.05). This datum can exclude any direct influences of the coating layer onthe mechanical strength of the specimens rather than an indirect effect on the maturation of the material.

### Scanning electron microscopy (SEM)

**Fig. 3:**
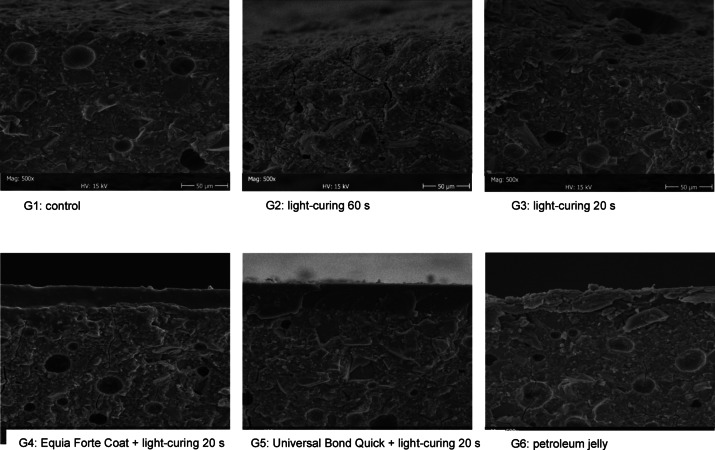
SEM micrographs (500× magnification) of cross sections as obtained after the flexural failure in representative specimens from each group (G1–G6) after aging for 28 days.

SEM images of representative cross sections of the flexural strength test specimens after 28 days in all groups revealed the internal structure of the material (Fig. 3). The images showed multiple fracture lines and air bubbles within the material. The coating thickness was also evaluated. Proprietary coat displayed a uniform layer of 25 μm, slightly lower than the manufacturer’s specifications (35–40 μm)^27^. The bonding agent exhibited a uniform thickness ranging from 30 to 35 μm. Both adhesive coatings were still clearly present after 28 days with no signs of degradation. The petroleum jelly showed a non-uniform surface layer in which petroleum was mingled with the HVGIC, with a mean thickness spanning from 15 to 25 μm.

### Water sorption and solubility

**Fig. 4:**
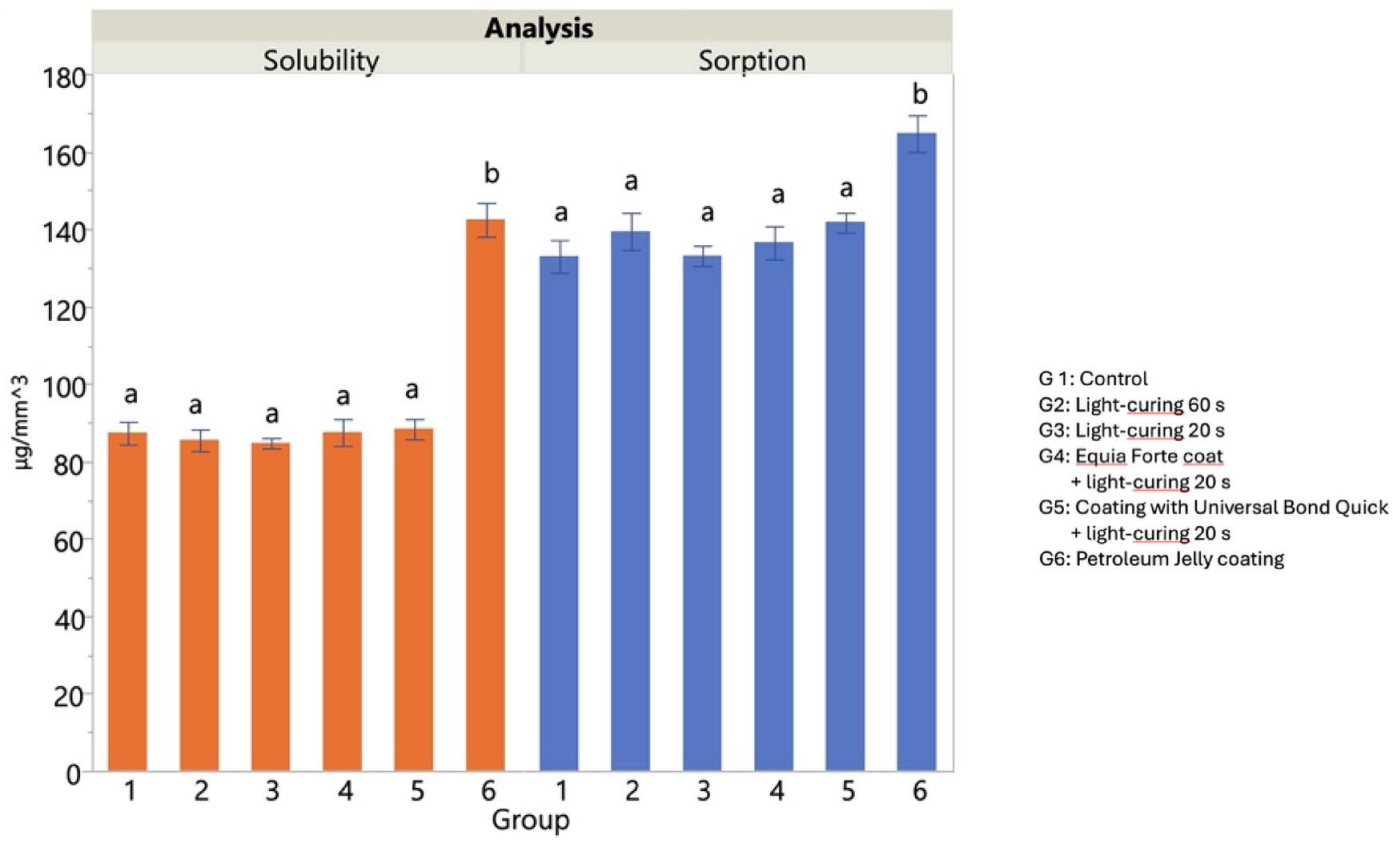
Water sorption and solubility for each group (1–6) after immersion in artificial saliva for 28 days. Means (µg/mm^3^) ± 1 standard error are displayed, while different superscript letters indicate a significant difference as assessed by Tukey’s, *p* < 0.05.

Measurements of water sorption and solubility are shown in Fig. 4. The values did not show significant differences (*p* > 0.05) comparing groups 1 to 5. The only treatment that differed from the control in terms of water sorption and solubility was petroleum jelly application (Group 6, *p* < 0.05).

### Fluoride release

**Fig. 5:**
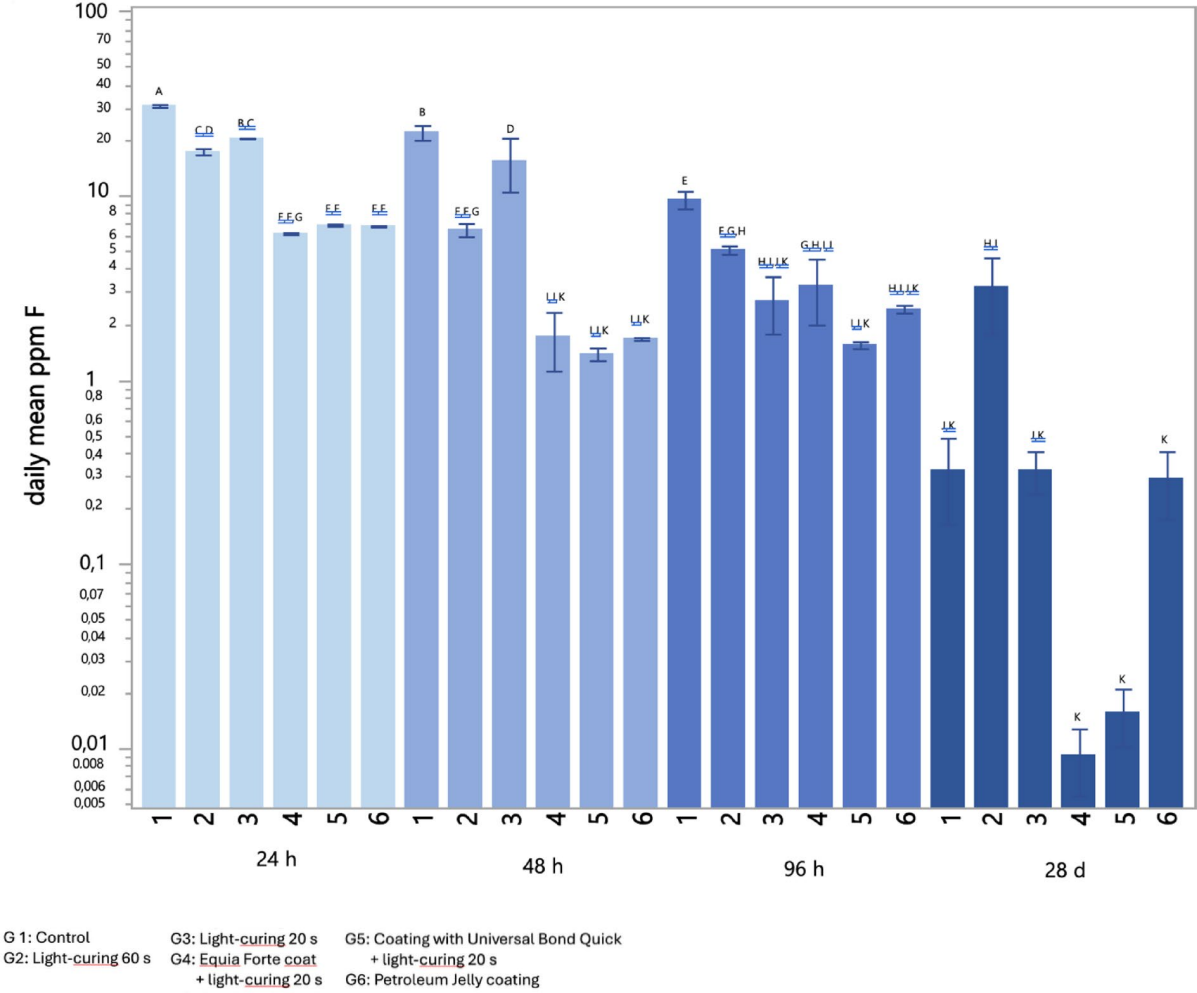
Fluoride release for each group (1–6) at 1, 2, 4 and 28 days. Daily mean (ppm F) ± 1 standard error are displayed, while different superscript letters indicate a significant difference as assessed by Tukey’s, *p* < 0.05.

In Fig. 5 the daily mean fluoride release of the specimens is presented at the tested time points. Fluoride release at 24 h was significantly lower in the groups subjected to surface treatment compared to the control (30.81 ± 0.94 ppm/day, *p* < 0.05). Specifically, all coated specimens (proprietary coat, bonding agent, and petroleum jelly) exhibited the lowest fluoride release values, 6.23 ± 0.16, 6.91 ± 0.24 and 6.81 ± 0.10 ppm/day (*p* < 0.05), respectively. The release profile remained consistent at 48 and 96 h, with all coated specimens exhibiting an overall lower daily fluoride release compared to the control group (*p* < 0.05). After 28 days, the groups treated with the proprietary coat and bonding agent showed significantly lower fluoride release values than the other groups (0.01 ± 0.01 and 0.02 ± 0.01 ppm/day, respectively, *p* < 0.05). Petroleum jelly and light-curing for 20 s showed similar fluoride release values to the control group (0.32 ± 0.42 ppm/day). Light-curing for 60 s showed the highest fluoride release values (3.20 ± 3.79 ppm/day, *p* < 0.05), displaying the most stable daily fluoride release over the tested time frame.

## Discussion

Flexural strength, which reflects fracture resistance, is a critical parameter for evaluating these materials, as restorations are subjected to shear and flexural stresses during masticatory loading, which are transferred to the tooth-restoration interface^28^. Three-point bending test is a widely used and clinically relevant method included in ISO standards for assessing the flexural strength of dental restorative materials^29,30^. However, there is currently no established reference in the literature for the minimum acceptable flexural strength of glass ionomer dental restorations. The most comparable guideline is provided by ISO 4049:2019, which defines standards for resin-based composite materials^27^. Given the similarity in mechanical challenges and functional loading, the Authors adopted the same testing protocol, including a flexural strength threshold of 80 MPa, to interpret the results of the present study.

Based on these results, the minimum acceptable strength threshold^27^ was not exceeded in all groups at 24 and 48 h. In fact, these values are consistent with many findings in the literature, which reports an average flexural strength of approximately 25 MPa for the uncoated material^14,28,31,32^. After 96 h, all groups except controls exceeded the threshold value, indicating that the material has reached a degree of maturity to withstand masticatory load. At 28 days, the flexural strength values for each group remained stable, indicating that the materials had reached a complete setting and maturation over time. Such findings are fundamental as insufficient flexural strength can contribute to early failure of restorations as a result of microcrack formation and adhesion deficiencies at the tooth-material interface. A statistically significant improvement in flexural strength was observed in the surface-treated groups compared to controls. According to the results of the three-point bending test, the untreated material reached flexural strength values exceeding the threshold approximately 5.5 days after placement. Clinically, these findings suggest that high-viscosity glass ionomer cement (HVGIC) restorations as the material tested should not be subjected to occlusal loading for approximately 5 to 6 days after placement, in absence of any further surface treatment. The latter significantly accelerated this process: both tested resin-based coats (Equia Forte Coat and Clearfil Universal Bond Quick) outdid the threshold in less than 2 days, petroleum jelly within 2.5 days, and light curing for 60 s within 3 days, representing a time reduction of 50% to 66%.

Equia Forte Coat (GC Europe) is a new-generation reinforced resin with nanofiller particles^13^, while Clearfil Universal Bond Quick (Kuraray Noritake) is a single-component, light-cured universal adhesive containing 10-MDP monomer^33^. Applied over the GIC restoration, they have a protective effect against extrinsic water, allowing the complete maturation of the GIC reaction with delayed water exposure. Coating infiltration enhances internal integrity by filling micro-porosities and reducing the risk of crack initiation^19,21^. These findings align with previous studies, showing enhanced mechanical properties of GICs when protected by surface coatings^18,19,28,34–36^. Petroleum jelly provides a comparable protective barrier during the early post-application phase^20,28^. Petroleum jelly use is a much more cost-effective possibility being advised in clinical practice by several studies^19,37^. To the best of our knowledge, the only study that analyzed petroleum jelly coating effect on the flexural strength was performed by Gorseta et al.^38^. They evaluated the influence of coating with either petroleum jelly or light-cured varnish and storage medium on the flexural strength of two GICs (Fuji Equia Fil and Ketac Molar Aplicap). Their results showed comparatively lower MPa values after 7 days than those observed in the present study after 4 days. Nevertheless, comparing the methodologies, there were slight differences in the artificial saliva composition and in the crosshead speed of the universal testing machine (1.0 mm compared to 0.5 mm). In fact, other differences had a much higher influence on the results, in particular the bar specimens dimensions (25 mm of length compared to 12 mm) and the material (Fuji Equia vs. Equia Forte HT). It must be acknowledged that increasing specimen’s length decreases the measured strength in an inverse ratio. Furthermore, the material tested by Gorseta et al. is a previous formulation of the GIC that has a presumably lower strength.

Light curing applied for 60 s to the surface of the restoration provides thermal energy, which accelerates the acid-base reaction responsible for the GIC setting^23^. Our results confirm a significant increase in flexural strength in the light-cured for 60 s group compared to the control group. High temperatures can also increase the powder-to-liquid ratio through liquid evaporation, thereby enhancing the material’s mechanical properties^39^. Heating the GIC during the setting phase has been shown to reduce microleakage and improve the marginal adaptation of the restoration^40,41^. Furthermore, the compressive strength of GICs has been shown to increase at temperatures above 37°C^42^. This consideration supports its use as one of the most effective and potentially cost-efficient treatments to enhance the properties of the tested material.

In this study, all specimen surfaces were coated, as immersion in artificial saliva required complete protection from fluid exposure. This approach aligns with clinical conditions, since the surfaces in contact with the cavity walls are not directly exposed to oral fluids. The primary purpose of the coating was to protect the material from moisture during the early stages of maturation rather than to improve its mechanical properties. Additional bar-shaped specimens were prepared with the coating applied only to the upper or lower surface, in order to assess whether the coating itself could have contributed to increased mechanical strength, rather than simply acting as a moisture barrier during maturation. Furthermore, if the coating layer had influenced the flexural strength, one would expect that specimens coated on one side and tested with the coated surface facing downward would exhibit reduced crack initiation compared with the opposite configuration. However, the obtained results excluded any direct mechanical contribution of the coating layer, as no significant differences were observed between specimens coated on one side (tested in either orientation) and the uncoated control group.

Water sorption and solubility of GIC specimens showed no significant differences between control and surface-treated groups. The only exception was the group treated with petroleum jelly, which exhibited increased values. This datum can be most likely attributed to residual petroleum jelly detaching from the specimens, thus interfering with weight measurements. These results contrast with the findings reported by Jafapour et al.^35^ and Hankins et al.^43^, who documented a reduction in both solubility and water sorption in specimens treated with nanofilled resin coating. Differences in the methodologies were identified between the studies and the ISO 4049:2019 specifications. In particular, in our study the specimens’ dimensions (6.0 × 2.0 mm, surface: 94.3 mm^2^, volume: 56.5 mm^3^, surface-to-volume ratio: 1.67) were slightly lower than ISO’s (15.0 × 1.0 mm, surface: 400.6 mm^2^, volume: 176.7 mm^3^, surface-to-volume ratio: 2.26). Specifically, a lower surface and, especially, surface-to-volume ratio may have decreased the water sorption and solubility, eventually leading to no observable differences between groups. It must be noted that the specimen’s dimensions in the present study were chosen because they more closely match the ones of a typical restoration cavity found in the clinical setting. Indeed, the mean tooth dimensions (7.0 mm for premolars; 10.0 mm for molars) and the clinical indications of the used material prevent an excessive extension. Another difference was due to the storage medium that in the present study was a composition of artificial saliva compared to water for analytical use. It must be noted that the electrolytic composition of artificial saliva more closely resembles the oral environment, and may provide an additional explanation for the found behavior. The incubation time was brought up to 28 days in the present study as opposed to ISO’s 7 days, to align with data obtained by fluoride release and flexural strength. A general increase in sorption and solubility values compared to the ones available in the literature^35,42^, as the incubation time was four times longer than the ISO specifications. As all GICs materials undergo the maturation phase, such extended storage time might better assess changes in sorption and solubility that may affect its properties.

Unsurprisingly, fluoride release from GIC was significantly reduced after the application of proprietary coat and bonding agents, both in the immediate days post-placement and after one month. Similar outcomes have been observed in prior investigations^43–45^. This reduction is attributed to the protective barrier formed by the coating, which limits the early dissolution of the immature surface layer and thus restricts ion diffusion^43^.

Fluoride release from GIC as well as from many release-based materials follows a characteristic pattern, beginning with an initial “burst” within the first 24 h, associated with the early acid-base setting reaction^46^. This early release has been shown to be beneficial for enhancing remineralization and reducing the residual bacteria viability in carious dentin^7^. It was well demonstrated that, after this initial phase, fluoride release declines sharply over the first week and stabilizes at a low, sustained level over the following 10–20 days^47^. A small but measurable amount of fluoride may continue to be released over the long term, potentially lasting for several months to years, contributing to the material’s cariostatic potential^46,47^. Our results confirmed such behavior.

Unexpectedly, in our experiment specimens subjected to 60 s light irradiation exhibited an increased fluoride release compared to controls after 28 days. Although the mechanism is not fully understood, heat may affect fluoride release by altering the glass-polyacid reaction dynamics or modifying the ions diffusion rate through the GIC matrix. Nevertheless, contrasting findings in the literature regarding this latter behavior highlight the need for further research^48–50^.

No simple correlation between fluoride release and water sorption or solubility was observed. Although fluoride release inherently requires some degree of water diffusion through the material, only limited elution of other ions is expected; thus, fluoride release alone likely contributes minimally to overall water solubility. Further improvements in material formulation aimed at maximizing fluoride release while maintaining solubility within the stringent ISO limits may help clarify these findings and should be considered a priority for future research.

The inclusion of a universal adhesive as a coating material was justified by its widespread use in clinical practice as an integral component of resin composite placement protocols. Moreover, given that manufacturer-provided coatings are substantially more expensive, this study sought to determine whether a more accessible and readily available alternative could provide comparable performance. However, the potential degradation of this adhesive layer in the oral environment remains uncertain. SEM analyses of specimens stored for up to 28 days revealed no evidence of surface degradation. Nevertheless, aging in artificial saliva cannot fully replicate clinical conditions, where erosion, abrasion, and attrition coexist with complex biodeterioration processes.

In fact, the main limitation of this study is related to its in vitro design, which does not fully replicate the complexity of the oral environment. In clinical conditions, variables such as masticatory forces, toothbrushing, fluoride-containing toothpastes, and fluctuations in temperature and pH values can significantly influence the outcomes. Therefore, further investigations under simulated oral conditions and correlation with the results of long-term clinical trials could be useful. Another potential limitation of the present study is the absence of an additional experimental group in which the coating was light-cured for a longer duration than that recommended by the manufacturer, specifically, resin-coated specimens irradiated for 60 s. Such a protocol might have allowed the material to benefit from both the protective effect of the coating and the additional heat generated during prolonged irradiation. However, it should be noted that manufacturers generally discourage extending the curing time of resin coatings or dentin bonding agents beyond 20 s, as this may negatively affect their physical and chemical properties. For this reason, extended light exposure was intentionally not combined with the application of a photo-curable coating material in the present investigation. It has nevertheless been demonstrated that thermal stimulation during the setting reaction of glass ionomer cements can reduce microleakage and improve marginal adaptation, suggesting that controlled heat application may represent a promising avenue for future studies^40^. Moreover, Gavić et al. demonstrated that the heat generated during light irradiation is not significantly transmitted to the bulk of the glass ionomer cement (GIC)^51^. This finding indicates that, even when a surface temperature rise occurs, as confirmed by the data of the present study, most of the heat dissipates rapidly, thereby preventing any detrimental thermal effect on the material. In addition, scanning electron microscopy (SEM) analysis revealed no increase in the number of microcracks in specimens subjected to prolonged irradiation compared with the control group.

In conclusion, this study demonstrated that all tested surface treatments, namely the proprietary coat, a universal bonding agent, petroleum jelly, or thermal treatment via light irradiation significantly enhanced the mechanical performance of a high-viscosity hybrid glass ionomer cement in terms of flexural strength. While untreated HVGIC required over 5 days to reach clinically acceptable strength values, the application of surface coatings accelerated this maturation process, reducing the required time by up to 66%. However, these surface treatments reduced fluoride release with the exception of light irradiation, and had no impact on water sorption and solubility. These findings are in favor of applying a coat to improve the longevity of the restoration and its resistance to occlusal stresses, while light irradiation may provide both enhanced mechanical properties and biological behavior in terms of fluoride release, also being a cost-effective possibility. The latter, however, may not be always possible in the clinical setting as it requires longer application times. Such considerations are expected to provide valuable ground for the clinicians in choosing the best way to improve the performances of this restorative material.

## Materials and methods

A total of 210 GIC specimens were prepared using custom-made ePTFE molds. A 2 × 2 × 12 mm mold was used to obtain 120 bar-shaped specimens (flexural strength measurements) while a 2 × 6 mm mold was used for 90 cylindrical specimens (water sorption/solubility and fluoride release). Each capsule containing the hybrid glass-ionomer cement (Equia Forte HT) was activated depressing the plunger and vibration-mixed in an amalgamator (HL-AH G5, Zoneray, Shanghai Dynamic Industry Co., Shanghai, China) for 10 s as per the manufacturer’s instructions, then immediately applied. The molds were slightly overfilled, covered with transparent Mylar strips, and compressed with a glass plate and a 100 g weight to minimize air bubble inclusion. The specimens were then left to harden for 3 min. Afterwards, the specimens were randomly divided into 6 groups (*n* = 20 bars / group and *n* = 15 cylinders / group), and subjected to different surface treatments: GROUP 1: control group, without surface protection; GROUP 2: light irradiated for 60 s at 1100 mW/cm^2^ (Valo, Ultradent Products, South Jordan, Utah, USA); GROUP 3: light irradiated for 20 s at 1100 mW/cm^2^; GROUP 4: coated on all surfaces with Equia Forte Coat and light cured for 20 s at 1100 mW/cm^2^; GROUP 5: coated on all surfaces with a bonding agent (Clearfil Universal Bond Quick, Kuraray Noritake, Tokyo, Japan) and light cured for 20 s at 1100 mW/cm^2^; GROUP 6: covered on all surfaces with petroleum jelly. Specimens subjected to light-curing were irradiated from all sides, and the output intensity of the curing unit was verified using a radiometer.

Additional bar-shaped specimens (5 per coating type) were prepared, with the coating applied only to either the top or bottom surface. This design aimed to rule out the possibility that the coating itself contributed to an increase in mechanical strength, rather than merely serving to protect the material from moisture during the maturation phase.

To test the thermal effect of the light-curing on non-coated specimens, an additional set of *n* = 10 cylindrical specimens was made as previously described, and the variation in the temperature of the specimens compared to the baseline (room temperature) was measured after placement of the material inside the mold and at the end of the irradiation time after 20 s (*n* = 5) or 60 s (*n* = 5).

All specimens were subsequently stored in artificial saliva at 37 °C.

The artificial saliva was prepared by mixing 100 mL of 150 mM KHCO_3_, 100 mL of 100 mM NaCl, 100 mL of 25 mM K_2_HPO_4_, 100 mL of 24 mM Na_2_HPO_4_, 100 mL of 15 mM CaCl_2_, 100 mL of 1,5 mM MgCl_2_, along with 6 mL of 25 mM citric acid, to replicate the average electrolytic composition of human whole saliva. The final volume was adjusted to 1 L, and the pH was brought to 7.0 by adding 4 M NaOH or 4 M HCl dropwise under continuous agitation^52^.

### Flexural strength

**Fig. 6:**
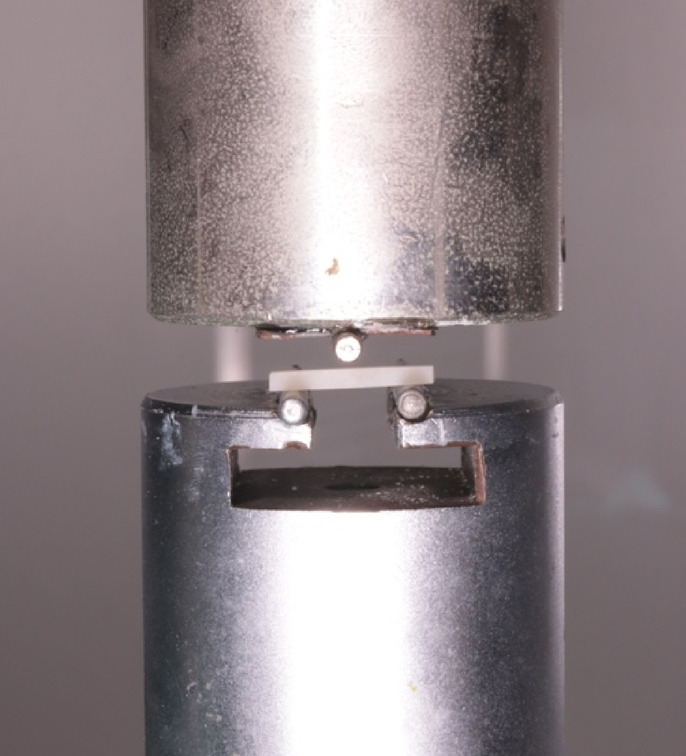
Universal mechanical testing machine including the setup for the 3-point bend test and a bar specimen positioned in the center of the testing area.

To assess the flexural strength (FS) of the GIC specimens, a 3-point bend test was performed, according to the ISO Standard 4049:2019. One hundred and twenty 2 × 2 × 12 mm bar-shaped specimens were obtained using custom-made ePTFE molds and, after setting, removed from the molds and randomly assigned to one of the six groups. After being treated on all surfaces as previously described, the specimens were stored in artificial saliva. After 24 h (T1), the first 30 specimens were measured with a precision caliper and then stressed using a universal mechanical testing machine (double-column 3300 Series Universal Testing Systems, Instron, IL, USA) at a crosshead speed of 0.5 mm/min (Fig. 6). The same procedure was repeated at 48 h (T2), 96 h (T3) and at 28 days (T4), each time on *n* = 30 specimens, *n* = 5 / group. The flexural strength was calculated using the following equation:

$$\sigma ~ = ~\frac{{3Fl}}{{2bh^{2} }}$$

where *F* is the load, *l* is the distance between the supporting rollers (= 8.0 mm), *b* is the specimen width, and *h* is the specimen height.

An additional set of bar-shaped specimens (*n* = 20) were obtained as previously described and treated with the proprietary coat as per GROUP 4, but only on one flat surface. Then, the specimens were stored for 24 h (T1) and half of them stressed with the coated surface upwards, while the other half with the stressed surface downwards. This additional test intended to ascertain that possible differences between Group 1 and 4 were not due to increased resistance caused by the layer of polymerized coat on the specimens’ surfaces.

### Water sorption and solubility

To assess water sorption and solubility, ISO 4049:2019 procedures were followed with modifications in the specimens’ dimensions, the storage medium and storage time. A total of 90 cylindrical specimens of 2 × 6 mm was obtained as previously specified. After setting and surface treatment (*n* = 15/group), they were first weighed using a precision balance at T0 (W1), stored in artificial saliva at 37 °C, and weighed again after 28 days (W2). They were then dehydrated in an oven at 60 °C for 24 h, and weighed once more at 29 days (W3). Water sorption and solubility values were calculated according to the following equations: Wsp = (W2 - W1)/V; Wsol = (W1 – W3)/V.

### Fluoride release

The fluoride release was assessed using a fluoride-specific electrode (Orion 9609-BN; Orion Research, Inc., Beverly, MA, USA) and digital ion analyzer (Orion 720 A; Orion Research, Inc.), previously calibrated with standard solutions (0.0625 to 1 or 2 to 32 µg F/mL), according to the fluoride concentration in the specimens expressed in µg F/cm^2^. The artificial saliva in which the 90 cylindrical specimens were stored was investigated at T1, T2, T3 and T4. A fluoride calibration curve and electrode drift adjustment were performed every half day, and the artificial saliva was replaced at each time interval. In addition, to decomplex fluoride ions, TISAB III (Total Ionic Solubility Adjustment Buffer, 940911, Orion Research, Inc.) was added to each tube. The mV value obtained was converted into ppmF using the calibration curve.

### Scanning electron microscopy (SEM)

Specimens from the flexural strength test, stored for 24 h in artificial saliva at 37 °C, were selected for SEM analysis. Cross-sections were examined using a scanning electron microscope (JEOL JSM-840 A, Tokyo, Japan) at magnifications ranging from ×50 to ×2000 to assess fracture patterns, failure modes, and coating thickness.

### Statistical analysis

The sample size was calculated based on the results of a pilot experiment on specimens stored for 24 h, using G*Power software (version 3.1.9.7, Heinrich-Heine-Universit¨at Düsseldorf, Düsseldorf, Germany). Considering the treatment as the primary source of variability, assuming a power of 0.80 and an alpha value of 0.05, we used flexural strength means and standard deviations of control and coated specimens to compute an effect size (d) of 1.72 that provided an estimated sample size of 5 specimens per each treatment group. Water sorption and solubility values of control and coated specimens obtained in a pilot experiment were used to calculate an effect size (d) of 0.93, assuming a power of 0.80 and an alpha value of 0.05. This analysis yielded an estimated sample size of 15 specimens per group.

Statistical analyses were performed with JMP 17.2 software (SAS Institute, Cary, NC, USA).

Shapiro-Wilk test and Levene’s test were performed to assess the normality of the distribution and the homogeneity of variances, respectively. One-way ANOVA followed by Tukey’s post-hoc test were performed to reveal statistically significant differences among the groups. A *p*-value < 0.05 was considered statistically significant. Survival and time required to reach a minimum required strength were analyzed by Weibull regression.

## Data Availability

Yes. The data is available in the manuscript body, or from the corresponding author upon reasonable request.
